# The association between adherence to a dietary approaches to stop hypertension (DASH) diet and neuro-psychological function in young women

**DOI:** 10.1186/s40795-021-00429-z

**Published:** 2021-06-09

**Authors:** Mansoore Saharkhiz, Zahra Khorasanchi, Samira Karbasi, Amir Masoud Jafari-Nozad, Mohsen Naseri, Mahtab Mohammadifard, Mahin Siami Ali Abad, Malaksima Ayadilord, Gordon A. Ferns, Afsane Bahrami

**Affiliations:** 1 Student Research Committee, Birjand University of Medical Sciences, Birjand, Iran; 2 Department of Immunology, Faculty of Medicine, Birjand University of Medical Sciences, Birjand, Iran; 3 Metabolic Syndrome Research Center, Mashhad University of Medical Sciences, Mashhad, Iran; 4 Cellular and Molecular Research Center, Birjand University of Medical Sciences, Birjand, Iran; 5 Infectious Diseases Research Center, Birjand University of Medical Sciences, Birjand, Iran; 6 Brighton & Sussex Medical School, Division of Medical Education, Falmer, Brighton, Sussex, BN1 9PH UK

**Keywords:** DASH diet, Insomnia, Depression, Stress, Quality of life

## Abstract

**Background:**

The adherence to a Dietary Approaches to Stop Hypertension (DASH) diet may have a bidirectional relationship with mental wellbeing. We aimed to evaluate the association between compliance with a DASH diet and neuro-psychological function in young women.

**Methods:**

In this cross-sectional study, a total of 181 girls aged between 18 and 25 years were recruited. The dietary intakes of study participants were evaluated using a valid and reliable food frequency questionnaire (FFQ) containing 65 food items. Neuropsychological function of participants was evaluated using standard questionnaires.

**Results:**

As may be expected**,** individuals in the highest tertile (T3) of adherence to DASH diet (highest adherence) were found to consume more folate, fruits, vegetables, low fat dairy, nuts, legume, and seed, less sweetened beverage and sodium, compared to the participants in the lowest tertile (T1, lowest adherence). There was a significant negative correlation between cognitive function and consumption of red and processed meat (r = − 0.168; *p* < 0.05); quality of life score with dietary sodium (r = − 0.151; *p* < 0.01) and depression score with dietary vegetables (r = − 0.174; p < 0.05). In multivariate multinomial logistic regression analyses adjusted for age, BMI and energy intake, adherence to a DASH-style diet was associated with a lower stress score (OR = 0.70; 95%CI: 0.34–1.47, *P* = 0.067; T3 vs. T1) and difficulty with sleep initiation (OR = 0.46; 95%CI: 0.21–1.00, *P* = 0.017; T3 vs. T1).

**Conclusion:**

Adherence to a DASH diet may be associated with reduced stress and difficulty with initiating sleep.

## Introduction

Impaired mental health comprises a wide range of conditions that affect the quality of life (QoL) and associate with an increased mortality rate in recent years. Considering the economic costs and the great burden they impose to the health system and individuals, prevention strategies for these conditions should be of significant importance [1].

In addition to genetic and environmental factors, diet has been suggested to play an important role in the development, and treatment of some psychological disorders. For instance, it has been reported that a diet rich in fish, folate and B-vitamins can alleviate the manifestations of depression. An established association was also shown between the dietary glycemic index (GI) and psychological diseases, depressive disorder, and mental functioning [2–4]. Diets containing of “junk foods” (high energy and low nutritional) have been shown to increase the risk of mental illness, but a healthy diet is associated with less depressive symptoms and can improve mood [5, 6]. Nutritionists are now emphasizing the importance of assessing dietary patterns, rather than single nutrients or foods, to better understand diet-related disease correlations.

The Dietary Approaches to Stop Hypertension (DASH) diet which was developed to reduce blood pressure, is characterized by high consumption of whole seeds, fruits, vegetables, leguminous plants and nuts, and only small amounts of low fat dairy, red meat, desserts, sweet and sugar-containing beverages [7]. Several studies have reported that a DASH diet has beneficial effects on different medical conditions, that include cardiovascular and renal disease [2, 3]. However, the existing data on the impact of the DASH dietary pattern on mental distress is still limited. In two previous studies, adherence to the DASH dietary pattern was reported to be inversely related to depression score [6, 8]. Furthermore, Torres et al. reported that a DASH-type diet could improve mood in postmenopausal women [9].

There have been few studies regarding to the effects of the DASH diet on mental health, particularly in young women, who are reported to be more prone to mental disorders such as depression and anxiety. We have therefore undertaken the current study to evaluate the relationship between adherence to a DASH diet and neuro-psychological function in healthy young women.

## Methods and materials

### Study design

In this cross-sectional study, 181 women were enrolled from 5 different universities in Birjand, South Khorasan, Iran in January 2020. The sample size was calculated to achieve 80% power and *α*′ = 0.05 (z value of 1.96) using data from a previous investigation (r = 0.24 for correlation between DASH score and depression) [10]. According to these assumptions a minimum of 140 participants was needed. Since we wanted to perform our project on a homogeneous population, to control for potential confounding factors, only unmarried, apparently healthy women, aged between 18 to 25 years were recruited. We excluded women with any established acute or chronic disease or those who were smokers. The Ethics Committee of Birjand University of Medical Sciences approved the study. All participants provided written informed consent.

### Dietary assessment

We used a validated semi-quantitative food frequency questionnaire (FFQ) to assess the food intake of subjects over the previous year [11, 12]. An experienced dietitian asked participants to describe their food consumption frequency for each item during the previous year on a daily, weekly, monthly, rarely or never basis. Food analysis was done using Diet Plan 6 software (forest field Software Ltd., Horsham West Sussex, UK). The DASH dietary pattern score was determined according to the method of Fung et al. [13]. The DASH score was calculated based on 8 food components such as: high intake of vegetables, fruits, legumes and nuts, whole grains and low intake of sodium, low-fat dairy products, red and processed meats, and sweetened beverages. For the composition of DASH score, values of 1 or 5 were assigned to each nutritional component using the quintiles as cut-off values. For vegetables, fruits, legumes and nuts, whole grains and low-fat dairy products the lowest quintile was scored 1 point and the highest quintile was scored 5 points. For red and processed meats, salt and sweetened beverages the scoring was inverted. Finally, the score of each group was integrated between a value of 8 (minimal adherence) to 40 (maximal adherence).

### General and clinical characteristics

Demographic and anthropometric data including age, parent death/divorce, height, and weight were collected in all participants using standardized protocols. Body mass index (BMI) was computed as weight in kilograms divided with height in meters squared [8].

### Neuropsychological analysis

#### Cognitive ability assessment (CAA)

The Cognitive Abilities Questionnaire (CAQ) is a valid and reliable tool which estimates 7 distinctive functions including: memory, inhibitory control and selective attention, decision making, planning, sustained attention, social cognition and cognitive flexibility [14]. This instrument is composed of 30 items, each that is rated on a scale of 1–5, to yield a total score ranging from 30 to 150. Higher scores reflect better cognitive performance [15]. A score below the median cut off for the cognitive abilities score was considered o be cognitive impairment.

#### Depression anxiety stress scales (DASS)

Depression, Anxiety and Stress Scale (DASS-21) is a valid and accurate tool to measure negative well-beings status [16]. This questionnaire consists of 21 items with 3 subscales (each consist of 7 questions) in which each item is rated on a 4-point Likert scale (0–3) to determine the severity of depression, anxiety and stress. Because DAS-21 is the brief version of DAS-42; the total score of each sub-class must be doubled. A higher score indicates more severe negative emotion. The validity and reliability of the DASS-21 has previously been validated for Iranian population [17]. Depression, anxiety, and stress scores were divided into two categories (No/Minimal state and some degree of disorder) according to the scores obtained for each subscale as follows: No (≤9) or some degree of depression (> 9); No (≤7) or some degree of anxiety (> 7); and No (≤14) or some degree of stress (> 14).

#### Quality of life (QoL)

The Short Form health survey (SF-12) derivative from the SF-36 is a widely reliable tool for measuring of physical and mental components of QoL. Validity and reliability from this tool has been established in Iranian population [18]. The SF-12 consists of 12 questions, covering 8 health domains, and higher scores show a better health dependent QoL [19]. A score below the median cut off for the QoL score was considered to be a low QoL.

#### Insomnia severity index (ISI)

The Insomnia Severity Index (ISI) is a brief self-report instrument which quantitatively measures severity of insomnia based on patient’s own perception. The questionnaire contains 7 question related to sleep disorder intensity, satisfaction with present sleep pattern, and anxiety-associated sleeping disturbance. Each item is rated on a zero to 4 measure (0 = no problem; 4 = many problem) to provide a total score ranging 0–28. Higher point indicates more degree of insomnia. Based on acquiring scores, individuals were categorized as: no clinically significant insomnia (0–7), mild to severe degree of insomnia (8–28). The Persian version of this tool with good reliability and validity for Iranian population was used in current study [20, 21].

#### Epworth sleepiness scale (ESS)

The Epworth sleepiness Scale (ESS) is an approved and validated simple method for measuring the general level of daytime sleepiness. The ESS is a brief, self-administered questionnaire estimates the probability of falling asleep in a variety of situations. ESS scores give a helpful measure of average sleep tendency, comparable to the results of all-day tests [22]. It consists of eight items with 4-point Likert scale (0–3) that requires the subject to estimate on a scale of 0–3 the frequency they have dozed in common daily situations. The sum of scores can range from 0 to 24 and higher scores specify worsen sleepiness [23, 24]. The severity of sleepiness was defined as: normal (score ≤ 10), mild to severe degree (score > 10).

#### Short sleep duration and difficult sleep initiating

Sleep signs were evaluated based on 2 asks, reporting to sleep during the past month: (1) “Do you have difficulty falling asleep at night?” (2) “How many times have you woken up early and, have trouble getting back to sleep?”. Short sleep duration was determined if a subject slept for fewer than 5 h/day, once or more a week [25]. Difficulty in sleep initiation was assessed as having problems falling asleep within half an hour once or more times per week [26].

### Statistical analysis

All statistical analyses were conducted by using SPSS, version 16 software packages. Data were evaluated for normality by applying the Kolmogorov-Smirnov test. One-way ANOVA test was used for comparison of continues variables with normal distribution across tertiles of DASH-style diet score. Depression, anxiety, stress, insomnia and sleepiness scores were divided into two categories (No/Minimal state and some degree of disorder) regarding to scores. Acquiring a score below median cut of QoL score was considered as low QoL. The correlation between component of the DASH diet score and neuro-psychological test scores was evaluated using Pearson correlation analysis. Multinomial logistic regression was used to evaluate the association between adherence to DASH diet and neuropsychological difficulties and we adjusted all variables for age, BMI and energy intake. A *P*-value < 0.05 was set as being significant.

## Results

### Demographic, anthropometric and clinical characteristics of the participants according to the DASH dietary pattern score tertiles

The mean age of the participants was 20.7 ± 2.2 years. The DASH diet scores were used to categorized the subjects into tertiles, with T1 being defined as the lowest tertile (least adherence; range: 12–22; *n* = 56), T2 (*n* = 63; range: 22–26), and T3 as highest tertile (highest adherence; range: 22–33; *n* = 62). There was no significant correlation among the demographic and anthropometric variables among the participants (*P* > 0.05; Table 1). But significant differences were found in BMI for participants in the 1st tertile compared to 3th tertile of adherence to the DASH dietary (*P* < 0.05).

**Table 1:** Demographic, anthropometric and clinical characteristics of the participants in different tertiles of the adherence to the DASH dietary pattern scores

Variables	T1 n = 56 (30.9%) range: 12–22	T2 n = 63 (34.8%) range: 22–26	T3 *n* = 62 (34.3%) range: 26–33	*P* value ^a^
Age (y)	20.8 ± 1.9	20.6 ± 1.5	20.9 ± 1.8	0.67
BMI (Kg/m2)	20.2 ± 2.3	20.4 ± 2.6	21.9 ± 3.4	**0.006**
Parent death, n (%)	3 (5.4)	1 (1.6)	1 (1.6)	0.36
Parent divorce, n (%)	2 (3.6)	1 (1.6)	3 (4.8)	0.60

Abbreviations: Body mass index (BMI)

Data presented as Mean ± SD

^a^ *p*-value from analysis of the variance (ANOVA) for groups comparison

### Dietary intakes of participants in different tertiles of the adherence to the DASH dietary pattern scores

The comparison between the mean of dietary intakes of participants in different tertiles of the adherence to the DASH dietary pattern scores are shown in Table 2. The intake of folate, fiber, fruits, magnesium, potassium, vegetables, nuts, legume, whole grain, seed and low fat dairy were higher among individuals in the highest tertile of adherence to DASH diet than others in the lowest tertile and the value of sweetened beverage and sodium were higher among subjects in the first tertile of adherence to DASH diet compared to the participants in the third tertile.

**Table 2:** Dietary intakes of participants in different tertiles of the adherence to the DASH dietary pattern scores

Variables	DASH diet adherence			
	T1	T2	T3	*P* value
DASH score, Median (IQR)	20 (17–21)	24 (23–24)	28 (27–29)	–
***Dietary nutrient intake***				
Total energy (kcal)	2170 ± 840	2166 ± 658	2177 ± 816	0.99
Carbohydrate (g/1000Kcal)	64.8 ± 39.5	57.5 ± 28.1	63.6 ± 31.2	0.54
Protein (g/1000Kcal)	35.3 ± 14.4	29.9 ± 14.9	31.9 ± 14.1	0.207
Fat (g/1000Kcal)	13.9 ± 12.8	16.5 ± 11.2	15.3 ± 11.2	0.54
Dietary fiber (g/1000Kcal)	5.9 ± 3.2	6.4 ± 3.1	8.0 ± 3.7	**0.006**
Zinc (mg/1000Kcal)	2.59 ± 1.39	2.23 ± 1.20	2.51 ± 1.10	0.28
Folate (μg/1000Kcal)	74.3 ± 38.2	81.7 ± 34.7	107.4 ± 40.9	**< 0.001**
Calcium (mg/1000Kcal)	447.9 ± 259.8	365.2 ± 217.5	466.2 ± 250.1	0.070
Magnesium (mg/1000Kcal)	160.1 ± 61.4	186.6 ± 96.7	219.6 ± 77.0	**0.001**
Potassium (mg/1000Kcal)	2112 ± 501	2412 ± 886	2814 ± 854	**< 0.001**
***Components of DASH-diet style***				
Fruits (serving)	1.4 ± 1.2	2.4 ± 160	2.8 ± 2.5	**0.001**
Vegetables (serving)	0.6 ± 0.6	1.1 ± 0.9	2.2 ± 1.0	**< 0.001**
Nuts, legume, seed (serving)	1.2 ± 2.3	1.8 ± 1.1	2.5 ± 2.1	**0.016**
Low fat dairy (serving)	1.7 ± 0.38	4.1 ± 0.5	7.3 ± 7.4	**< 0.001**
Whole grain (serving)	2.1 ± 0.5	3.0 ± 3.7	4.2 ± 4.7	**0.030**
Red and processed meat (serving)	3.0 ± 4.1	2.5 ± 2.1	2.3 ± 2.1	0.44
Sweetened beverage (serving)	1.1 ± 1.4	0.6 ± 0.7	0.4 ± 0.9	**0.004**
Sodium (mg/1000Kcal)	1727 ± 640	1512 ± 900.9	1272 ± 812	**0.018**

Data presented as Mean ± SD

†obtained from ANOVA test

### Correlation coefficient between neuropsychological tests and components of DASH score

The score range obtained was from 0 to 39 for depression, 0–30 for anxiety, 0–42 for stress, 0–28 for insomnia, 0–19 for daytime sleepiness, for cognitive abilities, 19–44 for QoL as well as 4–11 h for sleep duration. There was a significant negative correlation between total cognitive abilities score with red and processed meat (r = − 0.168; *P* < 0.05), QoL score with sodium (r = − 0.151; *P* < 0.01) and depression score with vegetables (r = − 0.174; *P* < 0.05). There was a significant positive correlation among QoL by nuts, legume, seed (r = 0.183; *P* < 0.05), insomnia with sweetened beverage consumption (r = 0.205; *P* < 0.01) and sleep duration with whole grain (r = 0.179; *P* < 0.05) (Table 3).

**Table 3:** Correlation coefficient between neuropsychological tests and components of DASH score

Food groups (serving)	Cognitive abilities	Depression	Anxiety	Stress	Quality of life	Insomnia	Daytime sleepiness	Sleep duration
	r	r	r	r	r	r	r	r
Fruits	0.031	−0.087	0.003	0.093	−0.105	−0.011	0.085	0.033
Vegetables	0.096	−0.174^*^	−0.146	−0.096	0.12	0.034	0.071	0.054
Nuts, legume, seed	0.028	−0.92	0.027	−0.060	0.183^*^	−0.063	− 0.003	0.005
Low fat dairy	0.005	−0.025	−0.070	− 0.054	0.062	− 0.012	0.004	− 0.054
Whole grain	0.019	0.055	−0.19	0.012	0.081	−0.097	−0.052	0.179^*^
Red and processed meat	−0.168*	0.027	0.112	0.084	−0.077	0.118	0.031	−.021
Sweetened beverage	0.046	−0.017	−0.002	0.059	−0. 127	0.205^**^	0.117	−0.071
Sodium (mg/1000Kcal)	0.013	0.148	0.021	−0.064	−0.151^**^	− 0.093	0.42	− 0.003

*p < 0.05

**p < 0.01

***p < 0.001

### Multivariate adjusted odds ratios (95% CIs) for neuropsychological disorders across tertiles of DASH-style diet scores

In multivariate multinomial logistic regression analyses adjusted for age, BMI and energy intake, adherence to DASH style-pattern was associated with a lower stress score (OR = 0.32; 95%CI: 0.14–0.71, *p* = 0.009; 2^st^tertile with 1^st^DASH tertile) and less difficulty in initiating sleep (OR = 0.46; 95%CI: 0.21–1.00, *p* = 0.017; third tertile versus first DASH tertile) (Table 4).

**Table 4:** Multivariate adjusted odds ratios (95% CIs) for neuropsychological disorders across tertiles of DASH-style diet scores

Variables	Tertiles of DASH score		
	T1 (n = 56)	T2 (n = 63)	T3 (n = 62)
Cognitive impairments	Ref.	1.07 (0.52–2.22)	0.62 (0.30–1.28)
Depressive mood	Ref.	0.90 (0.44–1.86)	1.6 (0.78–3.42)
Anxiety behavior	Ref.	0.53 (0.26–1.12)	1.54 (0.74–3.21)
Stress	Ref.	0.32 (0.14–0.71)**	0.70 (0.34–1.47)
Lower quality of life	Ref.	1.02 (0.42–2.44)	1.32 (0.56–3.02)
Insomnia	Ref.	0.81 (0.39–1.67)	0.89 (0.43–1.84)
Daytime sleepiness	Ref.	0.92 (0.43–1.99)	0.79 (0.37–1.73)
Short sleep duration	Ref.	0.51 (0.12–2.32)	0.70 (0.18–2.76)
Difficult sleep initiating	Ref.	0.67 (0.32–1.40)	0.46 (0.21–1.00)*

Tertile 1 was considered as reference group

Adjusted for age, BMI and energy intake

*p < 0.05

**p < 0.01

***p < 0.001

## Discussion

In the present study, we have shown that a high adherence to a DASH dietary pattern, characterized by high consumption of fruits and vegetable, as well as low intake of red and processed meat, sugar-containing beverages and sweets, is associated with lower odds of stress and sleep disturbance in young women.

Although the relationship between dietary patterns and mental health is unclear, it is possible that psychological stress and eating habits are related. Cartwright et al. showed that children who eat fatty foods and snacks frequently, and consume low amounts of fruits and vegetables, had greater levels of stress [27]. Excessive stress can affect the QoL and a previous study reported an inverse association between stress score and QoL among university students, those who face an increased risk of mental conditions due to different stressors [28].

Improvements in diet and lifestyle may have beneficial impacts on depression symptoms. We found that depression score was negatively associated with vegetables intake and this is in line with the findings of previous studies [29, 30]. A meta-analysis of eighteen studies with 446,551 participants has indicated that fruit and vegetable consumption is inversely associated with the risk of depression [29]. Additionally, a prospective cohort study reported that frequent use of vegetables can play a protective role against depression in the elderly [30].

The mechanisms by which vegetables, or ‘healthy dietary patterns, decrease the risk of depression is not completely clear [30], but some nutrients including B group vitamins, n-3 fatty acids and antioxidant have been thought to be related with depressive behavior [31]. Oxidative stress and inflammation can both increase depressive symptoms by their effects on neurons of the autonomic nervous systems. Folate and B group vitamins, with their beneficial effects on inflammation and oxidative stress, are necessary for neuronal function and their deficiency is associated with depression, anxiety and dementia [32]. Non-nutrient phytochemicals with antioxidant activities found in vegetables could be another probable mechanism for this improvement [30].

Nuts are a part of the human diet and are considered to be health-promoting nutrients. Our result showed that the consumption of nuts, legumes and seeds is associated with a higher QoL. These nutrient-rich foods contain substantial amounts of polyphenol antioxidants, macro- and micro-nutrients and other beneficial bioactive compounds which it’s intake decrease oxidative stress and inflammation, so can prevent several human disorders and increase the QoL [33].

We found an inverse relationship between sodium intake and QoL. Previous studies reported that excessive salt intake not only can lead to hypertension, but also plays an important role in endothelial dysfunction and cardiovascular and kidney disease progress as well as reduce life expectancy [34, 35]. Therefore, a diet containing low amounts of salt may be considered a potential strategy to control hypertension, decreases cardiovascular morbidity and improve the QoL.

We found a positive relationship between dietary whole grain intake and sleep duration which is consistent with the study of Grandner et al, who showed that short sleepers report higher intakes of protein and carbohydrate, but low dietary fiber consumption; altered sleep duration is probably associated with changes in the secretion of appetite-related hormones [36].

Diet plays an important role in cognitive function, especially in childhood and the growth period, due to providing the components for nerve formation and brain development [37]. In the current study, we have found an inverse relationship between cognitive abilities score and red/processed meat consumption. Consistently, it has been show that Western dietary pattern, characterized by high intake of processed foods, sweet and red meat, has been found to be associated with greater levels of mental distress in adolescents [38]. Additionally, a cross-sectional study conducted on the adult population in UK demonstrated that higher consumption of red meat is related to weaker cognitive functions such as reasoning ability and short-term memory [39].

Due to the importance of sleep on QoL and its impact on psychological wellbeing, it is essential to identify the main factors that may effect this cycle. Eating habits modification is a potential approach to prevent sleep complications. Rostami and co-researchers demonstrated that greater commitment to a DASH diet is associated with lower prevalence of insomnia [40]. Results of the present study indicated that sweetened beverage intake was associated with grater odds of insomnia. Caffeine, as an important active component of sweetened beverages, has been showed to be inversely associated with sleep duration and may disturb sleep, leading to difficulty falling asleep, insomnia, day time sleeplessness [41].

Indeed, one of the most significant findings of the current study was that adherence to a DASH-type diet decreased the likelihood of difficulty in initiating sleep. High intake of sweets can increase the glycemic response and induce oxidative stress. Long-term sugar intake has been showed to negatively affect brain serotonin 5-hydroxytryptophan receptor sensitivity in laboratory animal models [42, 43]. In another study, adherence to higher fat/lower carbohydrate diet was associated with a reduction in serotonin release in the hypothalamus of Wistar rats [44]. There is a high likelihood that the consumption of vitamins, minerals and fibers may improve sleep quality. Tryptophan is an essential precursor to serotonin syntheses, a neurotransmitter known for controlling the sleep cycle and inducing feeling of sleepiness. Group B vitamins are also required for serotonin synthesis. Therefore, a balanced and diverse diet that can provide fruits, vegetables and whole grains, like DASH, can improve sleep [45].

We showed that individuals with a greater adherence to a DASH-style diet had a higher intake of potassium, magnesium, and fiber. Because of the content of green leafy vegetables, nuts, seeds, legumes and whole grains, the DASH has a high amount of magnesium. Also, fruits and vegetables in the DASH diet provide a high content of potassium and fiber. Dietary fiber has low energy because it is resistant to breakdown by gastrointestinal enzymes, and fiber-containing foods i.e. whole grains, vegetables and fruit have a low energy density [46]. On the other hand, as is consistent with our results, Davison et al. reported that higher levels of energy (Kcal), fiber, B vitamins, calcium, phosphorus, potassium, and magnesium intakes were associated with better mental health [47].

To the best of our knowledge, this is the first study examining the correlation between adherence to DASH diet and neuropsychological functions in young women. But, the present study has a number of limitations. First, we used a cross-sectional design that cannot demonstrate causality. Difficult sleep initiating was evaluated using self-reported questionnaires which may be predisposed to bias. Moreover, a FFQ is an unreliable tool for assessing the sodium intake. Finally, we did not evaluate physical activity in our population.

## Conclusion

We found a significant relationship between adherence to a DASH diet and lower stress scores and difficult sleep initiating among young women. It seems that following a healthy diet pattern can decrease mental distress, and enhance quality of sleep. Further longitudinal studies are necessary to confirm these results with larger population.

## Data Availability

The datasets used and/or analyzed during the current study are available from the corresponding author on reasonable request.

## Abbreviations

DASH: Dietary Approaches to Stop Hypertension
FFQ: Food frequency questionnaire
QoL: Quality of life
PUFAs: Poly unsaturated fatty acids
GI: Glycemic index
SBP: Systolic blood pressure
DBP: Diastolic blood pressure
BMI: Body mass index
CBC: Complete blood count
CAA: Cognitive Ability Assessment
CAQ: Cognitive Abilities Questionnaire
DASS: Depression Anxiety Stress Scales
ISI: Insomnia Severity Index
ESS: Epworth Sleepiness Scale
DIS: Difficult initiating sleep
PQS: Poor quality of sleep
DMS: Difficulty maintaining sleep
TRP: Tryptophan
5-HT1A: Serotonin 5-hydroxytryptophan
